# Assessment of the Impact of Turbo Factor on Image Quality and Tissue Volumetrics in Brain Magnetic Resonance Imaging Using the Three-Dimensional T1-Weighted (3D T1W) Sequence

**DOI:** 10.1155/2023/6304219

**Published:** 2023-11-15

**Authors:** Eric Naab Manson, Stephen Inkoom, Abdul Nashirudeen Mumuni, Issahaku Shirazu, Adolf Kofi Awua

**Affiliations:** ^1^Department of Medical Imaging, School of Allied Health Sciences, University for Development Studies, Tamale, Ghana; ^2^Department of Medical Physics, School of Nuclear and Allied Sciences, University of Ghana, Accra, Ghana; ^3^Radiation Protection Institute (RPI), Ghana Atomic Energy Commission, Accra, Ghana; ^4^Radiological and Medical Sciences Research Institute, Ghana Atomic Energy Commission, Accra, Ghana

## Abstract

**Background:**

The 3D T1W turbo field echo sequence is a standard imaging method for acquiring high-contrast images of the brain. However, the contrast-to-noise ratio (CNR) can be affected by the turbo factor, which could affect the delineation and segmentation of various structures in the brain and may consequently lead to misdiagnosis. This study is aimed at evaluating the effect of the turbo factor on image quality and volumetric measurement reproducibility in brain magnetic resonance imaging (MRI).

**Methods:**

Brain images of five healthy volunteers with no history of neurological diseases were acquired on a 1.5 T MRI scanner with varying turbo factors of 50, 100, 150, 200, and 225. The images were processed and analyzed with FreeSurfer. The influence of the TFE factor on image quality and reproducibility of brain volume measurements was investigated. Image quality metrics assessed included the signal-to-noise ratio (SNR) of white matter (WM), CNR between gray matter/white matter (GM/WM) and gray matter/cerebrospinal fluid (GM/CSF), and Euler number (EN). Moreover, structural brain volume measurements of WM, GM, and CSF were conducted.

**Results:**

Turbo factor 200 produced the best SNR (median = 17.01) and GM/WM CNR (median = 2.29), but turbo factor 100 offered the most reproducible SNR (IQR = 2.72) and GM/WM CNR (IQR = 0.14). Turbo factor 50 had the worst and the least reproducible SNR, whereas turbo factor 225 had the worst and the least reproducible GM/WM CNR. Turbo factor 200 again had the best GM/CSF CNR but offered the least reproducible GM/CSF CNR. Turbo factor 225 had the best performance on EN (-21), while turbo factor 200 was next to the most reproducible turbo factor on EN (11). The results showed that turbo factor 200 had the least data acquisition time, in addition to superior performance on SNR, GM/WM CNR, GM/CSF CNR, and good reproducibility characteristics on EN. Both image quality metrics and volumetric measurements did not vary significantly (*p* > 0.05) with the range of turbo factors used in the study by one-way ANOVA analysis.

**Conclusion:**

Since no significant differences were observed in the performance of the turbo factors in terms of image quality and volume of brain structure, turbo factor 200 with a 74% acquisition time reduction was found to be optimal for brain MR imaging at 1.5 T.

## 1. Introduction

In magnetic resonance imaging (MRI), evaluation of structural changes in the brain to establish associations with neurological disorders mostly requires segmentation and volume estimation of brain tissues like gray matter (GM), white matter (WM), and cerebrospinal fluid (CSF) [1]. The most recommended pulse sequence for accurate segmentation and brain volume estimation in both clinical and research contexts is the three-dimensional T1-weighted turbo field gradient echo (3D T1W-TFE) pulse sequence [2, 3]. This is because of its ability to reveal important details about aging and various neurological and neuropsychiatric diseases, such as schizophrenia, Alzheimer's disease, Parkinson's disease, and multiple sclerosis [4]. In addition, the 3D T1W-TFE sequences also offer better gray-white matter contrast and high spatial resolution with whole brain coverage [5]. To acquire 3D volumetric data, a thick slab of tissue must be excited and a second phase encoding gradient must be applied in a high slice selection direction. However, the time required for acquisition is significantly increased by the requirement to execute phase encoding in two directions [6]. This results in an increase in the scan time required for 3D T1W-TFE in clinical applications, as opposed to 2D imaging with an associated increased possibility of motion artifacts [4]. Therefore, reducing MRI acquisition time without image degradation is still difficult [7].

The turbo factor, also known as echo train length (ETL), is the number of echoes per repetition time (TR). It is one of the most important sequence parameters that determine the acquisition time [8]. Typically, increasing the turbo factor reduces the acquisition time. Three-dimensional (3D) pulse sequences require substantially higher turbo factors to make up for the higher acquisition times due to the multiple, significant number of encoding steps in the phase and partition directions [9]. This is because, as the turbo factor becomes higher, more lines of *k*-space per repetition time (TR) are filled, consequently decreasing the acquisition time [10]. However, high turbo factors also have a significant impact on image quality. The late echoes produced by higher turbo factors are typically weak; nonetheless, they contribute to the overall signal, which often leads to a low contrast-to-noise ratio (CNR) and signal-to-noise ratio (SNR). Furthermore, edge blurring brought on by high turbo factors may degrade image quality [11], particularly with short TEs [9]. Depending on the turbo factor, the CNR can affect the delineation and segmentation of brain structures, which could potentially impact brain volume measurement reproducibility.

To the best of our knowledge, a very limited number of studies have been reported in the literature on the performance of turbo factors with 3D T1W-TFE pulse sequences in brain imaging. Chalavi et al. [12] conducted a study with two 3 T MRI scanners to optimize a set of volumetric brain protocols including 3D T1W-TFE using five different voxel sizes. Also, Uten et al. [5] and Yunyun et al. [2] carried out a related study using 3D T1W-TFE to examine how acceleration factors affect clinical 1.5 T MRI image quality and subcortical brain volume estimation. The purpose of the current study was to evaluate the influence of the turbo factor on image quality (i.e., CNR, SNR, and Euler number) and structural brain volume measurements (i.e., cerebral white matter, gray matter, and cerebrospinal fluid) while also taking steps to determine the optimal TFE factor for imaging the brain using the FreeSurfer equivalent protocol. Assessing the influence of TFE factors on image quality could help improve lesion detection, diagnostic efficiency, and appropriate management of cooperative and noncooperative patients during imaging.

## 2. Materials and Methods

### 2.1. Data Acquisition

The protocol for this study was approved by three Institutional Review Boards, namely, the Ghana Health Service Ethics Committee (GHS-ERC: 009/11/21), the University of Ghana Ethics Committee for Basic and Applied Sciences (ECBAS 075/20-21), and the University of Ghana Medical Centre (UGMC-IRB/MSRC/0003/2022). All study participants gave informed written consent prior to study entry.

Five healthy volunteers with a mean age of 30 ± 6 years and no history of neurological illnesses or head trauma underwent brain MRI scans on different days. The study was conducted on a 1.5 T MRI Philips scanner (Philips Medical Systems, PC, Best, Netherlands) equipped with a 16-channel receive and transmit head and neck coil.

The FreeSurfer software package was used for data analysis. However, the FreeSurfer analysis library does not currently have a dedicated analysis protocol for imaging data acquired from the 1.5 T MRI Philips scanner. Equivalent sequence parameters were therefore carefully selected to acquire high-resolution whole brain images containing isotropic signal values [13] that could be accurately analyzed using the FreeSurfer software package. A thorough visual inspection of the acquired images was performed to check for any pulse sequence or patient-related artifacts. Following the visual assessment, a sequence-related artifact was identified, and scan parameters were further adjusted to obtain images free from artifacts.

To investigate the influence of turbo factors on the volumes of various brain structures and image quality, participants were scanned with the optimized protocol using turbo factors 50, 100, 150, 200, and 225.

Table 1 shows the initial FreeSurfer equivalent protocol acquisition parameters and the adjusted (or optimized) protocol parameters used for the study. The sequence parameters FA, TE, TR, FOV, NSA, WFS, matrix size, and voxel size were controlled throughout the acquisition as the turbo factor was varied from 50 to 225. However, as the turbo factor was varied, other acquisition parameters were automatically adjusted and therefore could not be controlled.  Table 2 shows the different values of parameters depending on the turbo factor that were automatically adjusted.

### 2.2. Measurement of Brain Tissue Segments

From each participant's MR brain image acquired, the volumes of three brain structures were extracted from the input (i.e., sagittal 3D T1W-TFE) raw anatomical images using the automated FreeSurfer software (version 7.1.1) available online at https://surfer.nmr.mgh.harvard.edu/. This was achieved using the “recon-all” pipeline with default settings through cortical surface reconstruction processes [14] running on Intel Core i7 macOS Sierra. The “recon-all” pipeline processes included skull stripping, volumetric labeling, intensity normalization, white matter segmentation, surface atlas registration, surface extraction, gyral labeling, and statistics [15, 16]. In this study, the structural volumes extracted from the reconstructed images were cerebral WM, GM, and CSF. The statistics file, which is made available after “recon-all,” was used to extract the volumes using the asegstats2table [17]. asegstats2table is a command in the FreeSurfer software suite that is used to convert the output statistics from the “aseg” (i.e., automatic subcortical segmentation) process into a tabular format.

### 2.3. Image Quality Metrics

Images acquired with the optimized protocol were analyzed with the automated FreeSurfer software. Three quantitative image quality characteristics (including contrast-to-noise ratio (CNR), signal-to-noise ratio (SNR), and Euler number (EN)) were used to evaluate the quality of the MR images acquired.

The FreeSurfer software package computes the GM/WM/CSF tissue contrast-to-noise ratio for volumes using the ratio of differences in signal strength between two tissue types and background noise; it estimates SNR as a ratio of the mean signal intensity inside a given region of interest (ROI), which were GM, WM, and CSF, and the standard deviation of the pixel values outside that ROI. The EN was used to assess the accuracy of cortical surface reconstruction [12]. The EN was estimated as the total number of reconstructed objects in an image minus the total number of holes in those objects [18].

### 2.4. Statistical Analysis

The Statistical Package for the Social Sciences, SPSS software (version 20, IBM Corp., USA), was used to analyze the data. To assess variation across all subjects of the estimated tissue volumes, CNR, SNR, and EN among the images acquired at varying turbo factors, the one-way ANOVA test was used. Statistical significance was set at an alpha value of *p* < 0.05. The Pearson correlation test was also used to estimate the relationship between the turbo factors and scan time. Descriptive statistics was also performed on nonnormally distributed data sets, where applicable, and the results were summarized in terms of median and interquartile range (IQR) values.

## 3. Results

**Figure 1:**
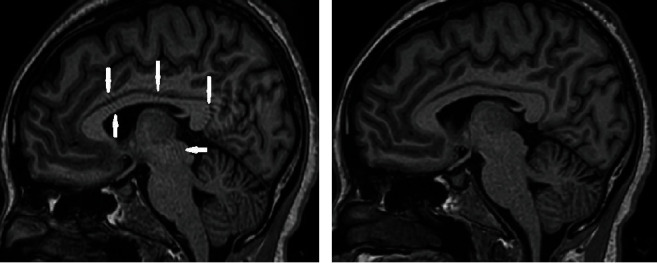
Sagittal 3D T1-weighted image of the brain (a) acquired with sequence parameters equivalent to FreeSurfer protocol: white solid arrows indicating areas of pulse sequence-related artifacts and (b) improved image acquired with the optimized protocol.

**Figure 2:**
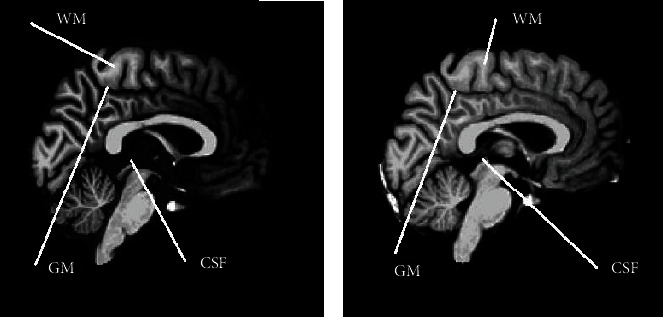
Segmented MR sagittal images after a “recon-all” pipeline from FreeSurfer free view: (a) acquired with turbo factor 50 and (b) acquired with turbo factor 225.

Figure 1 shows two sagittal MR images of the whole brain acquired from one of the volunteers with the 3D T1-weighted turbo field echo sequence using different pulse parameters at TFE factor 150. Moreover, the sagittal segmented images of the brain tissue probability maps showing cerebral white matter (WM), gray matter (GM), and cerebrospinal fluid (CSF) after “recon-all” are presented in Figure 2.

In Figure 2, the signal intensity in (b) appears to be higher as compared to that in (a), whereas the contrast between the tissues appears higher in (a) as compared to that in (b). The quantitative results on image quality and brain structural volume measurements from the five volunteers are presented in Tables 3–6.

From the study, no particular trend or variation was observed in the means of the SNR, CNR, Euler number (EN), and measured volumes as the turbo factor increased from 50 to 225. However, the SNR of the cerebral white matter, CNR of gray/white matter, CNR of gray/cerebrospinal fluid, and EN were highest for turbo factors 225, 50, 50, and 50, respectively. There were no statistically significant variations among the MR images acquired in the measured SNR of white matter (*p* = 0.93), CNR of gray matter/white matter (*p* = 0.85), CNR of gray matter/cerebrospinal fluid (*p* = 0.98), white matter volume (*p* = 0.99), gray matter volume (*p* = 0.99), cerebrospinal fluid volume (*p* = 0.99), and Euler number (*p* = 0.69) at the varying turbo factors. Moreover, the turbo factor and scan time were shown to have a highly negative and statistically significant Pearson correlation (*r* = −0.875, *p* = 0.05).

## 4. Discussion

The aim of this study was to optimize the 3D T1-weighted turbo field echo (3D T1W-TFE) pulse sequence while also finding the best TFE factor for brain imaging. Five TFE factors (i.e., 50, 100, 150, 200, and 225) on the MRI system were selected and applied in the acquisition of images. The effect on image quality of the range of TFE factors used in the acquisitions was evaluated through quantitative measurement of brain structures (i.e., the volumes of WM, GM, and CSF) and image quality metrics (i.e., SNR, CNR, and EN) using the FreeSurfer software suite (version 7.1.1).

From Table 5, it can be observed that turbo factor 200 has the highest median SNR (17.01), which is associated with the best signal-to-noise ratio. However, turbo factor 100 provides the most reproducible SNR, as it has the smallest interquartile range (IQR = 2.72). Turbo factor 50 produces the worst SNR and is the least reproducible turbo factor.

Similarly, turbo factor 200 has the highest GM/WM CNR median (2.29) and thus offers the best contrast-to-noise ratio. However, its reproducibility (IQR = 0.18) is better than only that of turbo factor 225 (IQR = 0.19); all other turbo factors show relatively better GM/WM CNR reproducibility than that of turbo factor 200. In addition to the highest GM/WM CNR, turbo factor 200 also offers the shortest patient scan time, which is a desirable factor in MR imaging.

For GM/CSF CNR, turbo factors 50 and 200 have the highest median values (1.15), associated with the best contrast. However, turbo factor 200 offers the least while turbo factor 100 offers the best reproducibility of GM/CSF CNR. Nevertheless, due to the longer scan time of turbo factor 100, it may not be optimal since a long scan time could cause patient motion associated with discomfort, which could consequently produce artifacts in the MR image.

Euler number (EN) refers to the quality of the input data (i.e., T1-weighted image). The smaller the EN, the better the tissue image displayed in the data. Turbo factor 225 has the lowest median EN (-21) followed by turbo factor 150 (-18). Turbo factor 200 does better on EN compared to only turbo factor 50. However, it shows better reproducibility in EN compared to turbo factors 100 and 220. Protocols with higher CNR, SNR, and lower EN values are considered to perform well, indicating good image quality [14, 19]. Overall, considering the SNR, CNR, and scan times, turbo factor 200 appears to offer better image quality compared to all the other turbo factors.

Signal-to-noise ratio (SNR), contrast-to-noise ratio (CNR), and Euler number (EN) are crucial metrics that define MR image quality [20]. High SNR is needed to identify tissue from noise in structural brain image studies [21]. While the Euler number is used as a measure of the input image quality, the segmentation of WM, GM, and CSF requires high CNR between GM and WM as well as between GM and CSF [22] and require high contrast-to-noise ratio.

No specific trend was observed in the variation of signal-to-noise ratio and contrast-to-noise ratio values as the turbo factors were increased. This is in agreement with the work conducted by Akhmad and Firmansyah [23] who found no particular variation in the SNR and CNR as the turbo factor increases. Moreover, no significant difference was observed in the performance of turbo factors in terms of both image quality and brain volume measurement. This may be due to the fact that whenever the turbo factor was changed on the MRI system, other sequence parameters (such as relative SNR, acquisition scan percentage, and minimum inversion time delay) were automatically adjusted to minimize any negative impacts that might affect the image quality. For instance, the scanner automatically adjusts the scan percentage to the nearest allowable number based on the operator's input for the turbo factor and some *k*-space manipulating parameters. In order to speed up image acquisition at the expense of spatial resolution, fewer *k*-space points are sampled within each encoded line. The key acquisition parameters that may change as a result of undersampling include the acceleration factor, matrix size, and FOV. To guarantee excellent image quality, the scan percentage should be kept between 80% and 100%. Aliasing artifacts will probably become too severe for values under 80% [24].

As was expected, the largest turbo factor (i.e., 225) produced the lowest CNR values for GM/WM, GM/CSF, and mean EN, whereas the smallest turbo factor (i.e., 50) produced the highest CNR values for these variables. On the contrary, it was observed that the largest turbo factor had the greatest SNR (i.e., 16.69) despite producing the lowest CNR and EN. As indicated by Akhmad and Firmansyah [23], this could be due to the individual biologically wide range of intensities. This variation should therefore be taken into account by the radiographer when determining the value of the turbo factor to be used for MRI scans of the brain, which will produce high SNR values.

Chalavi et al. [12] conducted a study to optimize the 3D T1W sequence with five different voxel sizes on two 3 T MRI scanners using sequence parameters equivalent to a FreeSurfer recommended protocol. After processing the acquired images with the FreeSurfer suit (version 4.5), the CNR ranged from 2.21 to 2.86 for GM/WM and from 0.69 to 0.94 for GM/CSF, while the mean EN ranged from -84 to -46. Their results were not consistent with those of the FreeSurfer sample data named “Bert” which were higher for GM/CSF CNR (1.09) but lower for GM/WM CNR (2.02) and mean EN (-49) [12]. However, the CNR of GM/WM, GM/CSF, and EN were improved after an adjusted protocol, indicating ranges from 2.6 to 2.96, 0.84 to 1.02, and -70 to -33, respectively [12]. By comparison, the mean GM/CSF CNR (i.e., 0.94 to 1.26) and mean EN (i.e., -23 to -13) in the current study appear to be higher with our optimized protocol than the reference values of Bert and that of the optimized protocol reported by Chalavi et al. [12]. On the contrary, the mean GM/WM CNR (i.e., 2.08 to 2.41) was higher than Bert but lower than the values obtained with the adjusted protocol. Although not too obvious, we may add here that the lower mean value of GM/WM CNR in this study could be attributed to differences in T1 relaxation properties between these tissues and contributions from field inhomogeneities due to variations in the susceptibility effects of different tissues.

Low SNR and CNR values do not entirely reflect poor data quality. A number of reasons such as motion, imperfect intensity inhomogeneity correction, strong background noise, some scanner artifacts, or a problem with the FreeSurfer analysis [13] could affect the image data quality. MR images acquired with a low SNR may seem a little grainy to the eye, particularly when contrasted with those acquired with a high SNR. A high SNR is not necessary to distinguish between two structures with high contrast differences, while a higher SNR is necessary to identify tissues with similar contrast [25].

The turbo factor is the number of refocusing pulses used during a single TR interval [26]. The effective TR for each line of *k*-space depends on the turbo factor selection. The effective TR decreases as the turbo factor increases, thereby shortening the time between successive RF pulses. Due to the shorter effective TR, there is less time for tissue relaxation and recovery, which can affect image contrast and SNR. A smart way to make sure that there are few to no time-wasting intervals between the pulses within the TR is to manipulate turbo factors with the goal of modifying the TR to a desired range of values [24]. For a 1.5 T system, decreasing the TR will result in an increase in the CNR of WM/GM [26]. The turbo factor does not alter the entire sequence design. However, it significantly affects TR, but the outcome of this influence must always be carefully considered because the lengthening of TR due to a turbo factor increase is frequently extremely large and occasionally may not even result in a benefit, even with the acquisition time reduction [24]. As can be observed in Table 5, there was a decrease in the mean CNR of GM/CSF, GM/WM, and Euler numbers as the turbo factor increased from 150 to 225 without a further reduction in the acquisition time.

Early echoes have a strong GM/WM contrast, while later echoes generate more off-resonance artifacts and decreased GM/WM contrast [22]. The extremely late echoes in 3D T1W sequences with high turbo factors have a low signal amplitude, and because the outer lines of *k*-space are filled with data from these echoes, the resolution is insufficient. Generally speaking, this causes image blurring that is frequently seen at the edges of tissues with various T2 decay rates. The impact may be mitigated by either decreasing the echo spacing and adding echoes to the echo train in a time-neutral manner [22], decreasing the size of the FOV in the phase direction, or selecting a wide receiver bandwidth [10] while limiting the number of sampling points to 64-128 [8]. Wide receiver bandwidths enhance the overall image quality by minimizing blurring, but it also lowers the SNR [10]. A wide receiver bandwidth, however, also lessens the impact of T2^∗^-induced signal decay, which can boost the SNR. The net impact on SNR is determined by the bandwidth, T2^∗^, and specific values of acquisition time [8].

Structural changes in the brain are frequently linked to neurodegenerative diseases, mental conditions, and healthy aging. Poor segmentation due to low image quality can significantly affect the measurement of brain anatomical tissues and, consequently, analysis of brain changes [27], resulting in overestimation or underestimation of brain volume. Amgad et al. [28] demonstrated changes in GM and WM volumes measured with two 3D T1W magnetization-prepared rapid gradient echo sequences (i.e., MP-RAGE and MP2RAGE). Besides patient motion, technical issues during the image acquisition process, such as magnetic field inhomogeneity, ghosting, or signal dropout, can create image artifacts [29]. These artifacts can obscure brain structures, alter tissue contrast, and affect the accuracy of brain tissue volume measurements [23]. The type of artifacts indicated by the solid arrows is known as truncation (or Gibbs ringing) artifacts. These artifacts are caused by insufficient acquisition of high spatial frequency data, typically in the phase encoding direction where undersampling is frequently employed to reduce scan time. The danger of these artifacts is that they can mimic the appearance of narrow structures such as a syrinx, the spinal cord, and intervertebral disks [30]. The best way to avoid truncation artifacts is to lower the thickness of the slices [8], by reducing the FOV or by increasing the matrix size [30, 31].

Fortunately, for this study, according to the one-way ANOVA test, there were no statistically significant differences (*p* < 0.05) observed between the individual volumes in the performance of the turbo factors varied. This means that the 1.5 T MRI scanner under investigation is capable of generating highly reproducible brain tissue volumes. This finding is in agreement with a similar study conducted by Uten et al. [5] who investigated the effect of the 3D T1W-TFE pulse sequence on the brain volume of subcortical structures. Our findings are also in agreement with that of Chalavi et al. [12] who indicated that, with the appropriate selection of pulse sequence parameters, the 3D T1W-TFE pulse sequence can be optimized to generate highly reproducible GM, WM, and subcortical volumes within and between MRI centers [12]. Reproducible brain tissue volumes are critical for accurate clinical diagnosis and patient follow-up studies. In longitudinal studies of neurological disorders, such as Alzheimer's disease, tracking changes in brain tissue volume over time is essential for understanding disease progression and evaluating treatment efficacy [32]. Generating reproducible brain tissue volumes in MRI scans, therefore, is crucial for accurate clinical diagnosis, reliable research findings, treatment planning, and understanding brain development and aging. It ensures the consistency and validity of the MRI data, making it an indispensable aspect of brain imaging and neuroscience research.

## 5. Conclusion

In this study, we have developed an optimized FreeSurfer equivalent 3D T1W-TFE pulse sequence protocol for a 1.5 T Philips MRI scanner that can eliminate artifacts from MRI images at all turbo factors. The optimized protocol for brain imaging was found to be operating at its optimal level as no significant differences were observed in the performance of the turbo factors in terms of image quality and structural brain volume measurements. However, turbo factor 200 appears to be the best for brain imaging, which reduced the acquisition time by up to 74% of the standard whole brain MR imaging time compared to turbo factor 50.

## Figures and Tables

**Table 1:** MRI scan parameters used for imaging.

Parameters	FreeSurfer equivalent protocol	Optimized protocol
Sequence	3D_T1W_TFE SENSE	3D_T1W_TFE SENSE
Voxel size (mm^3^)	1.00 × 1.00 × 1.00	1.05 × 1.05 × 1.10
FA (^o^)	7	7
TE (ms)	3.4	3.5
TR (ms)	7.6	7.5
Matrix size (mm^2^)	256 × 220	244 x 200
FOV (mm^3^)	256 × 256 × 160	254 × 237 × 180
Number of slices	145	145
Slice gap (mm)	0	0
Slice orientation	Sagittal	Sagittal
NSA	1	1
Acquisition WFS (pixel)/BW(Hz)	1.003/191.5	1.001/217
Acceleration factor (*P* = 1, *S* = 2)		

FA: flip angle; TE: echo time; TR: repetition time; FOV: field of view; NSA: number of signal averages; WFS: water fat shift; *P*: phase encoding; *S*: slice encoding.

**Table 2:** Parameters that were automatically adjusted as the turbo factor was varied.

Turbo factor	Time (min: sec)	Minimum TI delay (ms)	Scan percentage (%)	Relative SNR
50	20 : 25	219.4	88.1	1.04
100	10 : 13	404.6	88.1	1.04
150	05 : 08	589.4	66.1	1.20
200	05 : 08	775.1	88.1	1.04
225	05 : 08	867.9	99.1	0.98

**Table 3:** Signal-to-noise ratio of cerebral white matter and Euler numbers calculated from the various turbo factors for individual volunteers.

Volunteer	Turbo factor	SNR	Euler number		
			Left hemisphere	Right hemisphere	Mean
1	50	12.72	-28	-28	-28
	100	12.51	-32	-44	-38
	150	12.44	-22	-24	-23
	200	12.43	-44	-40	-42
	225	12.74	-34	-40	-37

2	50	19.98	-6	-6	-6
	100	18.74	-2	-6	-4
	150	20.4	-14	-4	-9
	200	20.04	-6	-8	-7
	225	19.44	-10	-22	-16

3	50	20.57	-6	-2	-4
	100	17.51	-12	-38	-25
	150	16.93	-26	-32	-29
	200	18.4	-12	-4	-8
	225	21.2	-16	-2	-9

4	50	14.91	-12	-18	-15
	100	15.83	-20	-10	-15
	150	16.41	-22	-14	-18
	200	15.09	-22	-16	-19
	225	15.73	-32	-10	-21

5	50	14.36	-8	-6	-7
	100	14.79	-10	-4	-7
	150	14.17	-8	-20	-14
	200	14.34	-14	-12	-13
	225	14.32	-36	-28	-32

**Table 4:** Contrast-to-noise ratio of WM, GM, GM/WM, and GM/CSF for both right and left hemispheres at different turbo factors.

Volunteer	Turbo factor	Left hemisphere					Right hemisphere				
		WM	GM	CSF	GM/WM	GM/CSF	WM	GM	CSF	GM/WM	GM/CSF
1	50	94.2 ± 10.0	64.1 ± 17.9	39.0 ± 16.6	2.152	1.063	94.7 ± 10.0	64.5 ± 17.9	39.4 ± 16.5	2.156	1.058
	100	94.8 ± 9.9	64.7 ± 18.0	39.1 ± 17.2	2.138	1.059	95.8 ± 10.5	65.4 ± 18.2	40.0 ± 16.9	2.099	1.044
	150	96.4 ± 10.2	66.3 ± 18.2	40.6 ± 17.1	2.089	1.061	95.6 ± 10.1	66.0 ± 17.8	40.8 ± 16.8	2.107	1.060
	200	95.1 ± 9.8	65.3 ± 18.0	40.8 ± 17.5	2.105	0.949	95.3 ± 10.4	65.4 ± 18.0	40.8 ± 17.0	2.069	0.996
	225	95.0 ± 9.8	66.2 ± 17.4	41.0 ± 16.8	2.074	1.078	95.4 ± 10.0	66.1 ± 17.6	41.6 ± 17.1	2.095	1.007

2	50	93.9 ± 9.4	62.5 ± 18.9	33.0 ± 16.6	2.215	1.366	93.2 ± 9.6	62.0 ± 18.8	33.3 ± 17.2	2.183	1.267
	100	94.4 ± 9.4	63.8 ± 18.6	34.6 ± 16.5	2.147	1.366	93.3 ± 9.7	62.8 ± 18.5	34.8 ± 17.1	2.136	1.240
	150	94.2 ± 9.0	64.2 ± 18.4	35.6 ± 16.8	2.141	1.315	94.5 ± 9.5	64.1 ± 18.6	35.9 ± 17.4	2.125	1.219
	200	94.9 ± 9.0	65.0 ± 18.3	36.3 ± 16.6	2.139	1.352	94.7 ± 9.3	64.8 ± 18.3	36.5 ± 17.1	2.120	1.274
	225	95.0 ± 9.4	65.5 ± 18.3	37.2 ± 16.5	2.075	1.317	94.0 ± 9.4	64.4 ± 18.3	37.3 ± 17.2	2.079	1.168

3	50	94.6 ± 9.0	63.2 ± 17.9	37.3 ± 16.3	2.448	1.150	93.5 ± 8.9	62.6 ± 17.7	37.0 ± 16.3	2.437	1.141
	100	95.0 ± 9.6	64.5 ± 17.8	38.6 ± 16.1	2.267	1.168	93.4 ± 9.2	63.4 ± 17.6	38.1 ± 16.0	2.292	1.124
	150	94.2 ± 9.4	64.4 ± 17.4	38.8 ± 15.6	2.264	1.199	95.2 ± 9.6	64.9 ± 17.7	39.1 ± 15.9	2.269	1.182
	200	95.6 ± 9.2	65.2 ± 17.7	39.0 ± 16.5	2.327	1.182	95.0 ± 8.8	64.5 ± 17.7	38.6 ± 16.9	2.397	1.113
	225	94.7 ± 8.7	64.6 ± 17.5	39.2 ± 16.4	2.379	1.130	94.9 ± 8.8	64.3 ± 17.7	38.9 ± 16.7	2.395	1.092

4	50	94.4 ± 9.7	63.0 ± 18.3	37.4 ± 17.6	2.310	1.017	93.5 ± 9.3	61.7 ± 18.4	35.9 ± 17.4	2.381	1.030
	100	95.9 ± 9.3	64.9 ± 18.1	38.9 ± 17.9	2.322	1.054	95.9 ± 8.8	63.7 ± 18.2	37.4 ± 17.7	2.397	1.074
	150	96.8 ± 9.5	66.2 ± 18.0	40.5 ± 18.2	2.252	1.007	95.2 ± 8.4	64.4 ± 17.9	39.0 ± 18.0	2.411	1.006
	200	94.0 ± 9.3	63.9 ± 17.7	39.5 ± 18.0	2.249	0.936	94.3 ± 8.8	63.6 ± 17.9	38.4 ± 17.8	2.362	0.997
	225	94.8 ± 9.0	65.9 ± 17.2	41.4 ± 17.8	2.23	0.983	93.4 ± 9.7	64.9 ± 17.2	40.4 ± 17.6	2.320	0.987

5	50	94.1 ± 10.0	61.5 ± 19.1	33.1 ± 17.8	2.274	1.191	93.4 ± 9.7	60.9 ± 19.1	33.1 ± 17.8	2.300	1.130
	100	94.7 ± 10.1	62.3 ± 19.1	34.2 ± 18.1	2.255	1.136	93.7 ± 9.6	61.5 ± 18.9	34.2 ± 18.3	2.310	1.074
	150	95.2 ± 10.0	63.0 ± 19.1	34.5 ± 18.5	2.237	1.141	94.7 ± 9.7	62.3 ± 19.0	34.2 ± 18.4	2.301	1.124
	200	95.3 ± 9.9	63.3 ± 18.6	34.6 ± 18.6	2.253	1.174	94.8 ± 9.5	62.7 ± 18.7	34.5 ± 18.5	2.336	1.144
	225	94.4 ± 9.6	63.6 ± 18.5	35.3 ± 18.3	2.173	1.178	94.9 ± 9.4	63.5 ± 18.8	35.9 ± 18.9	2.244	1.069

**Table 5:** Median, first quartile, third quartile, and interquartile values for SNR of white matter (WM) and CNR of white matter/gray matter (WM/GM) and gray matter/cerebrospinal fluid (GM/CSF).

Turbo factor	Scan time (mins)	SNR				CNR GM/WM				CNR GM/CSF				EN			
		Median	Q1	Q3	IQR	Median	Q1	Q3	IQR	Median	Q1	Q3	IQR	Median	Q1	Q3	IQR
50	20 : 25	14.91	14.36	19.98	5.62	2.29	2.20	2.35	0.15	1.15	1.06	1.16	0.10	-7	-15	-6	9
100	10 : 13	15.83	14.79	17.51	2.72	2.28	2.14	2.28	0.14	1.11	1.06	1.15	0.08	-15	-25	-7	18
150	5 : 08	16.41	14.17	16.93	2.76	2.27	2.13	2.27	0.14	1.13	1.06	1.19	0.13	-18	-23	-14	9
200	5 : 08	17.01	14.34	19.33	4.99	2.29	2.13	2.31	0.18	1.15	0.97	1.16	0.19	-13	-19	-8	11
225	5 : 08	15.73	14.32	19.44	5.12	2.21	2.08	2.28	0.19	1.11	1.04	1.12	0.08	-21	-32	-16	16

Note: Q1 = first quartile; Q3 = third quartile; IQR = interquartile range.

**Table 6:** Volumes of white matter, gray matter, and cerebrospinal fluid volumes at varying turbo factors acquired with the optimized protocol for each subject.

Subject	Turbo factor	Volume (mm^3^)		
		WM	GM	CSF
1	50	473413.00	637703.64	946.60
	100	472507.00	640707.39	912.30
	150	470672.00	643141.35	892.80
	200	475663.00	648423.83	885.60
	225	469797.00	644516.87	939.70

2	50	435088.00	560368.51	1416.00
	100	434852.00	564637.00	1402.30
	150	436659.00	564898.34	1469.70
	200	430452.00	567211.74	1397.50
	225	432302.00	572277.98	1457.00

3	50	448582.00	596951.33	984.30
	100	438942.00	601124.16	969.30
	150	436873.00	598663.98	943.00
	200	454293.00	594462.10	904.70
	225	450244.00	602944.02	990.30

4	50	489389.00	640700.02	1170.30
	100	482950.00	643332.20	1235.80
	150	485396.00	649465.12	1198.80
	200	486334.00	655381.61	1247.40
	225	487761.00	648293.15	1245.90

5	50	402319.00	532628.44	1042.00
	100	405824.00	540123.63	1012.00
	150	400198.00	540307.89	1009.60
	200	405641.00	533957.41	1041.20
	225	402229.00	540594.64	1058.10

## Data Availability

All data generated or analyzed during this study are included in this published article.
